# Achenbach Syndrome: Case Report and Discussion

**DOI:** 10.7759/cureus.23448

**Published:** 2022-03-24

**Authors:** Nicholas L Todd, Sean Bowling, Mallory Jengo, Micah Jones

**Affiliations:** 1 Medicine, Edward Via College of Osteopathic Medicine, Blacksburg, USA; 2 Orthopedic Surgery, LewisGale Medical Center, Salem, USA; 3 Orthopedics, Edward Via College of Osteopathic Medicine, Blacksburg, USA

**Keywords:** spontaneous digital hematoma, digital bruising, paroxysmal finger hematoma, idiopathic blue finger, achenbach syndrome

## Abstract

Achenbach syndrome is a rare, benign, self-limiting condition characterized by spontaneous, recurrent bruising of the digits without evidence of systemic disease or predisposing factors. We report a middle-aged Caucasian female that presented to the outpatient clinic with spontaneous bruising on the dorsal aspect of her left first metacarpal. Diagnosis of Achenbach syndrome was made through history and physical examination findings, and the patient was discharged with instructions to follow up as needed. It is important for providers to consider Achenbach syndrome to reassure patients and prevent an expensive or invasive workup.

## Introduction

Achenbach syndrome also called acute idiopathic blue finger or paroxysmal finger hematoma is a rare, benign medical condition in which patients present with acute painful bruising of at least one digit, classically sparing the fingertips [1]. It may occur spontaneously or in conjunction with minor injuries [2]. Achenbach syndrome was first cited by a German doctor, Walter Achenbach, in 1958, and few cases have been reported since its initial discovery [2,3]. Given the rarity of Achenbach syndrome, we believe it is important to document patient presentations to further illuminate this rare condition in the medical literature. We report a patient that presented to the outpatient clinic with recurrent spontaneous bruising of the first metacarpal.

## Case presentation

We present a 54-year-old Caucasian female with a past medical history of coronary artery disease, hypertension, rheumatoid arthritis (RA), dyslipidemia, gastroesophageal reflux disease, plantar fasciitis, and left first carpometacarpal (CMC) joint arthritis who presented to the outpatient orthopedic hand surgery clinic with a complaint of paroxysmal bruising of her left thumb. The patient was unable to remember when she first experienced symptoms or how often they recurred. She noticed that the bruising occurred sporadically and lasted three to seven days before completely resolving without treatment. The left thumb is the only location where the patient noticed bruising. Symptoms during exacerbations included pain, decreased grip strength, and a limited range of motion that significantly impaired her ability to perform her job as a food caterer. No prodromal symptoms were noted. She is a former smoker and quit 14 years prior. The patient received an autoimmune workup by rheumatology a year and a half prior to her visit, which was significant only for RA.

On physical exam, there was a 5 cm x 6 cm area of blue and purple bruising at the level of the radial styloid process that extended distally to the first metacarpophalangeal (MCP) joint of her left hand. Mild tenderness was noted over the left first dorsal compartment. Finkelstein’s test was negative, and extensor pollicis longus was intact. No paresthesia was noted in the left first metacarpal. Radial pulses were equal and strong in both wrists. Given that the patient recently underwent an autoimmune workup significant only for RA, a clinical diagnosis of Achenbach syndrome was made based on the patient’s history and physical exam findings. Other non-traumatic recurrent bruising conditions were considered, such as Raynaud’s phenomenon and psychogenic purpura; however, the symptoms described by the patient and physical exam findings were most consistent with Achenbach syndrome. She was counseled on the benign nature of this condition and was discharged from the clinic with instructions to follow up as needed.

## Discussion

**Figure 1:**
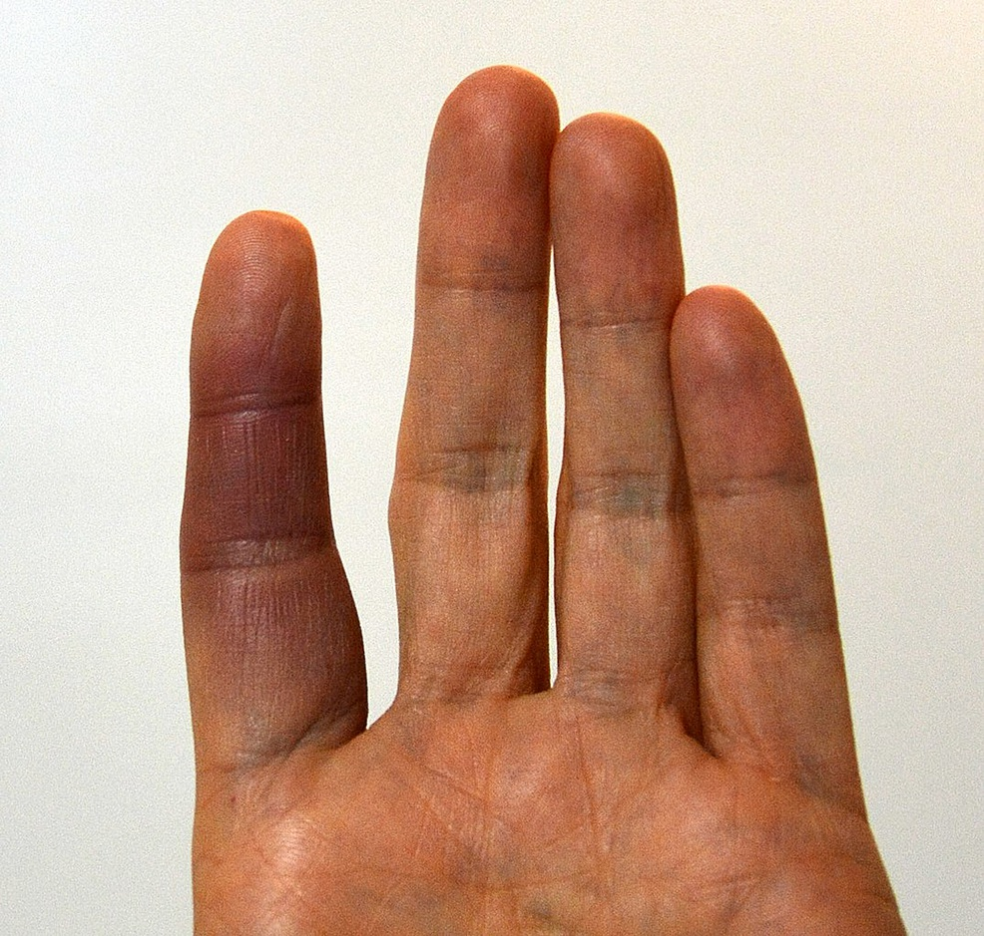
Achenbach syndrome Unedited image from Wikimedia Commons with permissions under license: https://creativecommons.org/licenses/by-sa/4.0/legalcode. The image is taken from https://commons.wikimedia.org/wiki/File:Paroxysmal_hand_hematoma_Achenbach_syndrome_doigt_bleu_04.jpg.

We present what we believe to be a classic case of Achenbach syndrome. The patient presented to the clinic with a history of recurrent spontaneous bruising on the dorsal aspect of her first metacarpal, which self-resolved after one week without treatment. Resolution of symptoms within this timeframe seems to agree with other reported cases of Achenbach syndrome, with some literature reporting an average symptom resolution time of four to seven days [1,2]. An example of Achenbach syndrome is demonstrated in Figure 1.

The differential diagnosis for a patient presenting with non-traumatic bruising of the hand includes conditions such as thromboangiitis obliterans, Raynaud’s phenomenon, psychogenic purpura, and acute limb ischemia. Associated diseases or precipitating events for the aforementioned conditions are outlined in Table 1. There are currently no known diseases or precipitating events associated with Achenbach syndrome, making this medical condition distinct from other disorders that cause digital discoloration. Currently, the only identifiable precipitating event associated with Achenbach syndrome is trauma, which has only been shown to occur in up to 30% of patients [2]. It is unclear, given current literature, if overuse (as in our patient who was employed as a food caterer) is associated with Achenbach syndrome.

**Table 1:** Differential diagnoses of digital bruising and their associated disease or precipitating event

Differential diagnosis for bruised fingers	Associated disease/Precipitating event
Thromboangiitis obliterans	Smoking
Raynaud’s phenomenon	Autoimmune disorders, cold temperatures
Psychogenic purpura	Anxiety, stress
Acute limb ischemia	Arterial embolism
Achenbach syndrome	None reported/Not established

In a retrospective study of 24 patients diagnosed with Achenbach syndrome, a demographic analysis showed that 83.3% of the patients were females with a mean age of 48 years [2]. Of these, 16.6% were smokers, 0% used alcohol, and 4.1% had a positive family history of digital bruising [2]. This limited data suggests the only established risk factors are age and female gender.

Capillaroscopic evaluation of patients with Achenbach syndrome demonstrated severe microhemorrhages in affected digits [4]. Since this finding is nonspecific, diagnosing Achenbach syndrome can be achieved by history and physical examination. Doppler sonography is an inexpensive and noninvasive tool that can be utilized to rule out more concerning etiologies such as emboli in unclear presentations. Results of the study demonstrate normal blood flow [2].

Achenbach syndrome may be misdiagnosed as a primary Raynaud’s phenomenon in many cases. Raynaud’s phenomenon, however, is a distinct disorder with different pathogenesis. Primary Raynaud’s phenomenon has been shown to be an exaggerated vasospastic response due to cold or emotion [5]. The thumb is generally spared in primary Raynaud’s phenomenon, and there are no capillary nailfold abnormalities, unlike Achenbach syndrome [6]. Secondary Raynaud’s phenomenon due to connective tissue disease demonstrates evidence of tortuous or hemorrhagic nailfold capillary loops [6].

Although Achenbach syndrome is rare, it is important for providers to understand this medical condition as a potential etiology of paroxysmal finger discoloration. Sudden-onset painful digital bruising can be a mentally traumatic event for patients; therefore, providers should offer reassurance to reduce anxiety and build rapport in these circumstances once the diagnosis has been made. Additionally, providers can spare their patients an invasive and expensive workup searching for the cause of bruising when a clinical diagnosis suggests Achenbach syndrome. Kordzadeh et al. created an algorithm for providers to distinguish Achenbach syndrome from other similar conditions [2]. As demonstrated in the treatment algorithm, it is imperative that providers rule out acute limb ischemia or other more sinister causes before considering Achenbach syndrome [2]. Future studies should be directed toward exploring risk factors, treatment options, or a potential genetic link to Achenbach syndrome.

## Conclusions

Achenbach syndrome is a benign, self-limiting digital bruising disorder that may elicit anxiety from patients when they first experience symptoms. It is distinct from many other causes of finger discoloration such as Raynaud’s phenomenon and is not currently associated with any inciting incidences or disease processes. It is important for providers to understand this medical condition as a potential etiology for paroxysmal finger bruising to prevent an extensive workup. The prognosis for Achenbach syndrome is good as there are no known complications or sequelae.
